# The immunosuppressive effect of glucocorticoids in human primary T cells is mainly mediated via a rapid inhibition of the IL-2/IL-2R signaling axis

**DOI:** 10.1186/s12964-025-02266-0

**Published:** 2025-06-04

**Authors:** L. Tatiana Albarracin Melo, Nekruz Abdulkhakov, Irina Han, Ali El-Bizri, Monika Brunner-Weinzierl, Burkhart Schraven, Luca Simeoni

**Affiliations:** 1 Institute of Molecular and Clinical Immunology, Medical Faculty, University Hospital, Otto-Von Guericke University, Magdeburg, Germany; 2 Health Campus Immunology, Infectiology and Inflammation (GC-I3), Medical Faculty, University Hospital, Otto-Von Guericke University, Magdeburg, Germany; 3 Department of Experimental Pediatrics, Medical Faculty, University Hospital, Otto-Von-Guericke-University, Magdeburg, Germany

**Keywords:** Glucocorticoids, T cells, Immunosuppression, IL-2 signaling, TCR signaling, Jak/STAT pathway, Erk1/2 activation, IκB activation

## Abstract

**Background:**

Glucocorticoids (GCs) are highly effective anti-inflammatory drugs that suppress T-cell activation, cytokine production, and T-cell proliferation. Nevertheless, at which molecular level and how fast GCs exert their immunosuppressive effect in T cells still remains elusive, as inconsistent genomic and non-genomic mechanisms of action have been proposed. One model postulates that GCs quickly inhibit proximal T-cell receptor (TCR) signaling via a non-genomic mechanism, whereas others have shown a strong inhibition of interleukin-2 (IL-2) transcription at later stages of T-cell activation. Due to their therapeutic significance, we have decided to shed light onto this issue and investigated how fast and at which level GCs inhibit T-cell activation by analyzing TCR and IL-2 signaling.

**Methods:**

We utilized primary human T cells isolated from healthy donors, which were stimulated with immobilized CD3/CD28 antibodies. These cells were treated with three different GCs, diflorasone, dexamethasone, and prednisolone.

**Results:**

Analyses of signaling kinetics revealed that GCs did not affect early TCR signaling as suggested by the normal phosphorylation levels of lymphocyte-specific protein tyrosine kinase (Lck), zeta-chain-associated protein kinase 70 (Zap70), linker for activation of T cells (LAT), and unchanged Ca^2+^ influx. Conversely, we found that GCs strongly and rapidly suppressed the activation of the Janus kinase (Jak)/ signal transducer and activator of transcription (STAT) pathway within 4–6 h upon CD3/CD28 stimulation in primary human T cells. This observation was in line with a strong inhibition of cytokine production and with the impaired upregulation of the IL-2 receptor (IL-2R) upon GC treatment, thus resulting in the abrogation of T-cell proliferation.

**Conclusions:**

Our study, by showing that GCs rapidly suppress the IL-2/IL-2R expression and signaling without significantly affecting proximal TCR signaling, has highlighted a clear mechanism of action of GCs that contributes to their therapeutic efficacy.

**Supplementary Information:**

The online version contains supplementary material available at 10.1186/s12964-025-02266-0.

## Introduction

Glucocorticoids (GCs) are lipid-soluble steroid hormones produced by the adrenal cortex that participate in the regulation of a plethora of physiological processes, including cell proliferation, differentiation, apoptosis, migration, and metabolism [1, 2]. GCs can be also synthesized chemically and are widely used as immunosuppressive agents in clinical applications for the treatment of autoimmunity, allergy, cancer, and to prevent rejection following organ transplantation [3, 4]. GCs exert their effects upon binding to ubiquitously expressed glucocorticoid cytosolic receptors (GCRs), which in turn translocate to the nucleus, bind to the glucocorticoid response elements in the promoter region of target genes, thus activating or repressing gene expression. Despite their ubiquitous expression, the functional outcomes of GCs differ by cell type. The cell-type specific effect of GCs activity is determined by different factors including the expression of co-regulatory proteins, the interaction with other transcription factors, post-translational modifications, and, most importantly, by non-genomic mechanisms of action [2, 5, 6]. In fact, in addition to their well-known genomic mode of action, GCs also induce rapid cell responses within minutes, which cannot be regulated at the transcriptional level. Different non-genomic mechanisms of action have been proposed including modulation of signaling pathways and of mitochondrial functions [7–10].

Due to their immunosuppressive properties, the mechanisms of action of GCs in immune cells and in particular in T cells have been deeply investigated during the past years [2, 5, 8, 11–13]. Despite this intense research, it is not yet clear how GCs suppress T-cell responses and in particular, proliferation. Both genomic and non-genomic mechanisms of action have been proposed. Early studies suggested that GCs inhibit IL-2 production at later stages of T-cell activation, between 20 and 48 h upon stimulation [14–17]. Defective IL-2 production has been suggested as a key factor in the impaired T-cell proliferation caused by GCs. A subsequent study reinforced these findings by demonstrating that dexamethasone inhibits the expression of IL-2 receptor (IL-2R) and Janus kinase 3 (Jak3), thereby disrupting IL-2 signaling in T-cell blasts 24 h after stimulation [18]. Additional research identified another potential late-stage immunosuppressive effect of glucocorticoids, characterized by the enhanced expression of the inhibitory receptor programmed cell death protein 1 (PD-1) on activated human and murine T cells [19, 20]. In addition to the previously mentioned effects on gene expression, which were primarily observed at later stages of T-cell activation, further studies have shown that GCs also exert rapid (within minutes), non-genomic effects during the initial stages of T-cell activation. Data published about two decades ago showed that a short treatment of human T cells with the synthetic glucocorticoid dexamethasone reduced the phosphorylation of the tyrosine kinases Lck and the related Fyn, thereby impairing the initiation of TCR signaling [21, 22]. Conversely, another study demonstrated that dexamethasone increased Lck activation in resting T cells [23]. Finally, research conducted using effector T cells described an additional non-genomic effect of GCs involving cytoskeleton rearrangements leading to diminished migration and impaired interaction with antigen-presenting cells (APCs) [24]. Although the available data do not define a clear immunosuppressive mechanism by which GCs inhibit T-cell activation and proliferation, a plausible scenario can be proposed to reconcile the existing findings. If GCs inhibit TCR signaling at the onset of T-cell activation (e.g. by inhibiting Lck or T cells/APCs interactions), this would in turn lead to defective IL-2 secretion and impaired CD25 upregulation at later stages, as both processes rely on TCR signaling. Thus, GCs may inhibit T-cell activation and proliferation (i) by disrupting TCR signaling and (ii) by transcriptionally repressing IL-2/IL-2R expression, as demonstrated by earlier studies.

In this study, we performed a detailed characterization of the still unknown immunosuppressive mechanisms of diflorasone, a glucocorticoid used as an anti-inflammatory compound for the treatment of skin disorders since many years [25–28]. More recently, diflorasone has also been proposed for the treatment of non-alcoholic fatty liver disease [29]. Furthermore, we compared the mechanisms of action of diflorasone with those of the well-characterized dexamethasone and prednisolone. Surprisingly, we did not observe an effect of GCs on the initiation of TCR signaling, as previously proposed. Conversely, we found that all three GCs tested in our study rapidly inhibit cytokine production and the upregulation of the expression of the IL-2R, thus impairing Jak/signal transducer and activator of transcription (STAT) signaling in primary human T cells.

In summary, our findings show that in primary human T cells, the immunosuppressive effects of GCs are not dependent on proximal TCR signaling. Instead, GCs exert their primary effect by rapidly inhibiting the IL-2/IL-2R expression and consequently Jak/STAT signaling, thereby suppressing T-cell activation and proliferation.

## Materials and methods

### Reagents

Diflorasone was purchased from MedChemExpress (New Jersey, USA). Dexamethasone and prednisolone were obtained from Sigma-Aldrich (St. Louis, MO, USA). All glucocorticoids were dissolved in dimethyl sulfoxide (DMSO) in a 10 mM stock solution and stored at −80°C. Phorbol 12-myristate 13-acetate (PMA) and ionomycin (Sigma Aldrich) were diluted in DMSO to 1 mg/ml and stored at − 20 °C.

### T-cell isolation

Density gradient on Pancoll (PAN Biotech, Aidenbach, Germany) was used to isolate human peripheral blood mononuclear cells (PBMCs) from heparinized blood samples collected from healthy volunteers. Human T cells were purified by negative selection using the Pan T cell isolation kit (Miltenyi Biotec GmbH, Bergisch Gladbach, Germany). The purity of the isolated T cells was > 95%. Cells were suspended in RPMI 1640 medium (PAN Biotech, Aidenbach, Germany) supplemented with 10% fetal bovine serum (FBS, Sigma-Aldrich, USA) and 2 μg/ml ciprofloxacin (Fresenius Kabi Austria GmbH, Austria). The study was approved by the local ethics committee (No. 175/18), and all blood donors provided written informed consent.

### Proliferation assay

For proliferation experiments, T cells (10^5^ cells/100µL) were incubated in 96-well culture plates (Corning Inc., Germany). The plates were coated with CD3 (UHCT1, 10µg/mL, Biolegend) and CD28 (28.2, 2.5 µg/mL, Biolegend) monoclonal antibodies. 1 μM of diflorasone, dexamethasone, prednisolone, or DMSO as a vehicle control was added directly to the wells at the beginning of the experiment. Additionally, decreasing concentrations of diflorasone from 1µM to 1 × 10^–8^ µM were tested in parallel to determine the half-maximal inhibitory concentration (IC50). Cells were plated in triplicates and cultured for 72 h at 37 °C. [^3^H]-thymidine (TdR) was added at 0.2 mCi/well for the last 7–8 h of the incubation time. At the end of the incubation period, cells were harvested, and radioisotope incorporation was measured using the beta plate liquid scintillation counter MicroBeta (Wallac, Turku, Finland).

### T-cell activation

T cells (1 × 10^6^/sample) were seeded in 24 well-plates (Corning Inc., Germany) coated with anti-CD3 (10 µg/mL) and anti-CD28 (2.5 µg/mL). Diflorasone, dexamethasone, or prednisolone (1 µM) were added to the media at the beginning of the experiment. The plates were incubated at 37 °C for 24 h. Subsequently, T cells were washed with phosphate-buffered saline (PBS) and incubated with anti-CD25-PE (M-A251, BD Biosciences) or anti-CD132-APC (TUGh4, Biolegend), anti-CD45RA-APC (5H9, BD Biosciences), anti-CD8-PE-Cy7 (HIT8, BD Biosciences), anti-CD4-FITC (L200, BD Biosciences) at 4 °C for 20 min in the dark. After incubation, cells were washed with PBS and analyzed using an LSRFortessa I (BD Biosciences, USA). The data were analyzed using FlowJo software (BD Biosciences, USA).

### Generation of T-cell blasts

T-cell blasts were generated as previously described [30]. Briefly, PBMCs were stimulated with plate-bound anti-CD3 and anti-CD28 antibodies for 48 h. After washing, the cells were rested in fresh medium for 24 h. Diflorasone, dexamethasone, or prednisolone (1 µM) were added to the medium for 2 h prior to stimulation with 100 U/mL recombinant IL-2 for 20 min. Cell lysates were then prepared and analyzed by immunoblotting.

### T cell lysates and immunoblotting

Human T cells (1.5 × 10^6^ to 4.5 × 10^6^) were treated with diflorasone, dexamethasone, or prednisolone (1 µM) for 2 h at 37 °C. Subsequently, cells were stimulated with biotin anti-CD3 (UCHT1, Biolegend) and biotin anti-CD28 (28.2, Biolegend) immobilized on sulfate latex beads 4% (Invitrogen, USA) or SuperAvidin™ Coated Microspheres (Bangs Laboratories Inc, USA) for the indicated time points, as previously described [31]. T cells were lysed in 1% lauryl maltoside, 1% NP-40, 1 mM phenylmethylsulphonyl fluoride, 1 mM Na_3_VO_4_, 10 mM NaF, 10 mM EDTA, 50 mM Tris–HCl (pH 7.5), and 150 mM NaCl. Samples were assayed by sodium dodecyl sulfate‒polyacrylamide (SDS-PAGE). Proteins were transferred using a semi-dry method onto a nitrocellulose membrane (Amersham Protran, USA). Membranes were blocked in 5% milk and incubated with primary antibodies in 5% bovine serum albumin (BSA) in tris-buffered saline (TBS)/0.2% Tween 20 for 1 h at room temperature (RT) or overnight at 4 °C. Blots were probed with the following antibodies: pY416 Src, pY319 Zap70, pY171 LAT, pT202/Y204 Erk1/2, pS32/36 IκB, pY694 STAT5, pY705 STAT3, pY980/981 Jak3 and IκB (Cell Signaling Technology, Danvers, MA, USA), as well as with Lck, Zap70, LAT, STAT5, STAT3, and Jak3 (Santa Cruz Biotechnology, Dallas, TX, USA), and β-actin (Sigma-Aldrich, Munich, Germany). Secondary antibodies coupled with horseradish peroxidase (Dianova/Jackson ImmunoResearch Laboratories, Inc., USA) were diluted in 5% milk and incubated for 1 h at RT. Target proteins were detected by ECL (Amersham/GE Healthcare; Chicago, USA). The data were processed and analyzed using the ImageJ software (freeware, http://rsb.info.nih.gov). The total median values of densitometric analysis were used for quantifications.

### Calcium flux

Human T cells were incubated with Indo-1 (Thermo Fisher Scientific, Waltham, USA) at 37 °C for 45 min in RPMI 1640 medium without phenol red (Gibco Thermo Fisher Scientific, USA) supplemented with 10% FCS. Subsequently, cells were washed and incubated with biotinylated CD3 and CD28 antibodies at 4 °C for 20 min. Diflorasone, dexamethasone and prednisolone (1 µM) were present during the whole process. Before measurement, a baseline of 1 min was recorded, after which 20µg/mL neutravidin (Thermo Fisher Scientific, Waltham, USA) was added for T-cell stimulation. As control for Indo-1 loading, cells were treated with 100 nM ionomycin (Sigma-Aldrich, Saint Louis, USA) 10 min after antibody stimulation. Calcium influx was evaluated utilizing an LSRFortesa II flow cytometer (BD Biosciences, USA) equipped with a helium cadmium laser emitting at 325 nm. Emitted light ranging between 395 and 500 to 520 nm was quantified, and the ratio of the two emission intensities was analyzed using FlowJo 10 software (FlowJo, LLC, USA).

### Apoptosis

Apoptosis was assessed using the Annexin V-FITC Apoptosis Detection Kit with 7-aminoactinomycin D (7 AAD) (Biolegend). T cells were treated with diflorasone, dexamethasone, and prednisolone at 1µM and stimulated in 24-well plate coated with and-CD3/28 for 24-48 h. Subsequently, cells were rinsed with PBS and resuspended in 1 × binding buffer at 1 × 10^6^/100 µL. After 15 min of incubation at RT with 5 µL of Annexin V-FITC and 5 µL of 7 AAD, 400 µL of binding buffer was added. Cells were then analyzed by flow cytometry using an LSRFortessa I (BD Biosciences, USA). Analyses were conducted using FlowJo 10 software (FlowJo, LLC, USA).

### Cytokines production

Human peripheral blood T cells were treated with diflorasone (1µM) for 2 h at 37 °C and stimulated with anti-CD3/28 immobilized on sulfate latex beads 4% (Invitrogen, USA) for 6 h at 37 °C. Subsequently, culture supernatants were harvested and frozen at − 80 °C. CD3/28-stimulated T cells treated with brefeldin A served as a negative control. Analysis of the supernatants was carried out using the MACSPlex 12 Kit human (Miltenyi Biotec, Germany) according to the manufacturer's instructions. Flow cytometry analysis was performed using LSRFortessa I (BD Biosciences, USA), and data were analyzed using FlowJo 10 software (FlowJo, LLC, USA).

### mRNA expression analyses

Human peripheral blood T cells were treated for 2 h with GCs as described above and subsequently stimulated with CD3/CD28 antibodies immobilized on microbeads for 6 h. RNA was isolated from pelleted cells with NucleoSpin RNA Isolation Kit (Macherey–Nagel). The cDNA was synthesized using revertAid H minus first strand cDNA synthesis kit (Thermo Fisher Scientific) and stored at –20 °C. Primer pairs to investigate the gene expression profile of IL-2 and CD25 (IL-2R) by real-time PCR (quantative (q) PCR) were purchased from TIB MOLBIOL. Gene expression was analyzed using Maxima SYBR Green qPCR Master Mix (Thermo Scientific) on a CFX96 Real-Time PCR detection system (Bio-Rad). Fold change in the expression of target genes was normalized to the expression of the housekeeping gene GAPDH.

Primer-Seq.:

- GAPDH rev CATggAATTTgCCATgggTgg.
- GAPDH fwd CggATTTggTCgTATTgggCg.
- IL-2R rev TTTCCCATggTggAggTTCC.
- IL-2R fwd CTCTgCCACTCggAACACAA.
- IL-2 rev gCCTTCTTgggCATgTAAAA.
- IL-2f wd TgCCACAATgTACAggATgC.

### Statistics

Data analysis was performed using Microsoft Excel for data organization and GraphPad Prism version 10.2 for statistical analysis and graphical representation. For comparisons involving more than two groups, repeated measures one-way ANOVA was used in combination with Dunnett's post hoc test. In experiments with two independent variables (e.g. treatment type and time), a two-way ANOVA was conducted followed by a Šídák post hoc test. We acknowledge that the small sample size (*n* = 3) limits the statistical power and robustness of the one-way or two-way ANOVA used in some figures. However, we chose to retain the statistical analysis to provide a preliminary indication of potential differences between conditions. Moreover, we show all individual data points to ensure full transparency.

## Results

### Diflorasone blocks T-cell proliferation

**Fig. 1:**
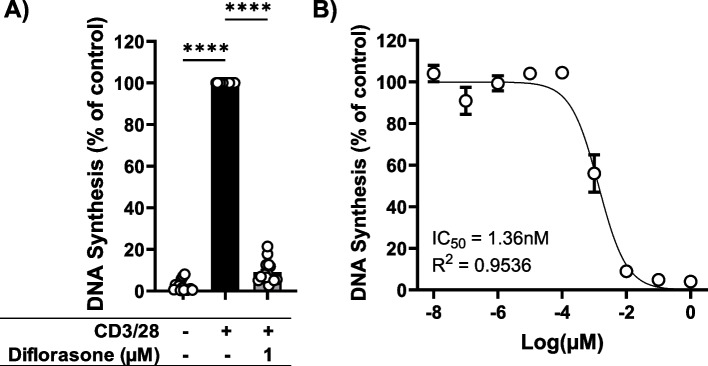
Diflorasone inhibits T-cell proliferation. Freshly isolated peripheral blood T cells were left either untreated or stimulated with CD3/CD28 antibodies. **A** T-cell proliferation was determined by [^3^H]-TdR incorporation assay in the presence or absence of diflorasone. Data are presented as the mean ± SEM of 15 independent experiments (15 donors). Statistical analysis was performed with one-way ANOVA followed by Dunnett’s post hoc test (**** *p* ≤ 0.0001). **B** The drug-response curve was determined by [^3^H]-TdR incorporation assay using a concentration range of diflorasone from 1 to 1 × 10^–8^ µM. Data are presented as the mean ± SEM of 4 independent experiments (4 donors). The IC50 value was obtained by nonlinear regression analysis using GraphPad Prism 10

To investigate the effects of diflorasone on T-cell functions, we initially evaluated its impact on the proliferation of peripheral blood human T cells. To this aim, T cells were stimulated using plate-bound CD3 and CD28 antibodies. After a 72-h incubation at 37 °C, T-cell proliferation was assessed using the [^3^H]-TdR incorporation assay. Diflorasone treatment blocked the proliferation of T cells stimulated with CD3/28 (Fig. 1A). Additionally, we cultured human T cells with different concentrations of diflorasone for 72 h to determine the IC50, which was 1.36 nM (Fig. 1B). The effect of diflorasone on proliferation was not due to increased cell death, as shown by the Annexin V/7 AAD assay (Figure S1).

### Diflorasone does not inhibit early TCR-mediated signaling events

**Fig. 2:**
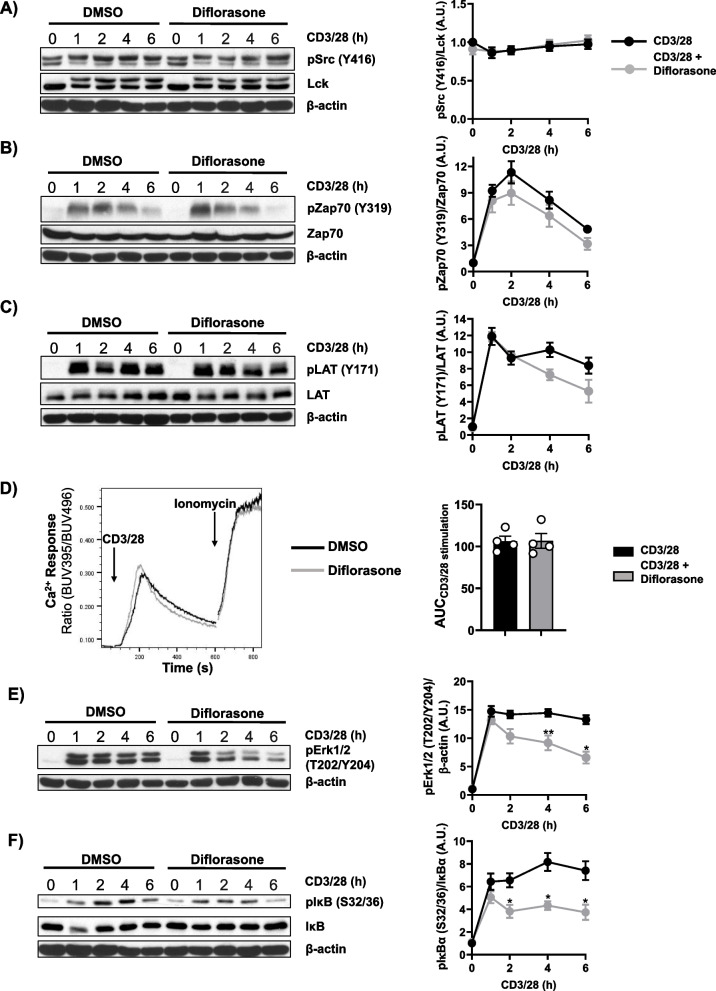
Diflorasone treatment does not affect TCR signaling but inhibits sustained Erk1/2 and IκB phosphorylation in CD3/CD28-activated human T cells. Freshly isolated peripheral blood T cells were pretreated with 1µM diflorasone for 2 h and subsequently stimulated with CD3/28-coated beads for the indicated time points. The phosphorylation levels and the expression of Lck **(A)**, Zap70 (**B**), LAT (**C**), Erk1/2 (**E**), and IκB (**F**) were assessed in cell lysates using phosphospecific, total protein or β-actin antibodies. For the detection of Lck phosphorylation, we used the phospho-Src antibody recognizing the highly conserved across Src-family kinases Y416. β-actin was used as a loading control. One representative immunoblot is presented on the left side of the figure (n = 4–6 donors). Bands in (**A**), (**B**), (**C**), (**E**), and (**F**) were quantified by normalizing the signal of the phosphorylated molecule to the signal of the corresponding total protein or β-actin and quantification analyses are presented in the graphs shown on the right side. Statistical analyses were performed using two-way ANOVA followed by Šídák´s post hoc test (*p ≤ 0.05 and **p ≤ 0.01). **D** Ca^2+^ influx was evaluated by flow cytometry upon CD3/28 stimulation of human T cells, which were pre-treated with diflorasone for 2 h. Ionomycin was employed to demonstrate equal loading with indo-1. A representative experiment is shown on the left side (*n* = 4 donors). The bar graph on the right side shows the quantification of the Area Under the Curve (AUC) of the CD3/28-stimulate samples

The effects of diflorasone on T-cell proliferation described above can either be due to the modulation of gene transcription, the most well-known mechanism of action of GCs, or to inhibitory effects on proximal TCR signalling. Indeed, it has been shown that dexamethasone and prednisolone inhibit the phosphorylation of Lck and the initiation of TCR signalling [21, 22]. To assess whether diflorasone also inhibits Lck and TCR signalling, human peripheral blood T cells were treated with diflorasone and subsequently stimulated with CD3/CD28 antibodies immobilized on microbeads, as previously described [31]. The data depicted in Fig. 2A show that diflorasone treatment does not affect the phosphorylation of Lck on the activatory Y394 and hence, we assumed that the activation of Lck is not affected by diflorasone. In agreement with this hypothesis, we did not observe significant differences in the phosphorylation of Y319 in the Syk-family kinase zeta-chain-associated protein kinase 70 (Zap70), an Lck downstream substrate, upon diflorasone treatment (Fig. 2B). In addition, we also did not observe a major effect of diflorasone on the phosphorylation of the transmembrane adaptor protein linker for activation of T cells (LAT), a Zap70 substrate (Fig. 2C) as well as in the rapid TCR-mediated Ca^2+^ influx (Fig. 2D). Taken together, these findings suggest that diflorasone, contrary to the effect observed for other glucocorticoids [21, 22], does not block the proliferation of human peripheral blood T cells by affecting the TCR-mediated Lck-Zap70-LAT-Ca^2+^ signalling cascade.

### Diflorasone inhibits the IL-2/IL-2R signalling axis

Conversely to the signalling pathway investigated above, whose activation depends on TCR triggering, other signalling pathways, such as mitogen-activated protein kinase (MAPK) and nuclear factor kappa B (NFκB), are activated by both the TCR and cytokine receptors (e.g. IL-2R). Whereas the TCR contributes to activation of these pathways at early time points (e.g. 1–2 h upon stimulation), cytokine receptors are likely responsible for their sustained activation at later time points upon T-cell activation [32]. Indeed, when we investigated the phosphorylation of extracellular signal-regulated kinase 1/2 (Erk1/2) and inhibitor of kappa B (IκB) as indicators of MAPK and NF-κB signalling, respectively, we found that, in contrast to molecules of the TCR signalling machinery whose activation decreases 1–2 h after stimulation (Fig. 2B-C), the phosphorylation of Erk1/2 and IκB remains sustained up to 6 h (Fig. 2E-F). The data presented in Fig. 2E-F show the effects of diflorasone on the phosphorylation of both Erk1/2 and IκB. We did not observe differences in their activation between diflorasone-treated and untreated T cells 1 h after T-cell stimulation. Conversely, diflorasone-treated T cells failed to sustain the activation of both Erk1/2 and IκB (Fig. 2E-F). These findings suggest that diflorasone primarily affects signalling pathways beyond those directly initiated by the TCR.

**Fig. 3:**
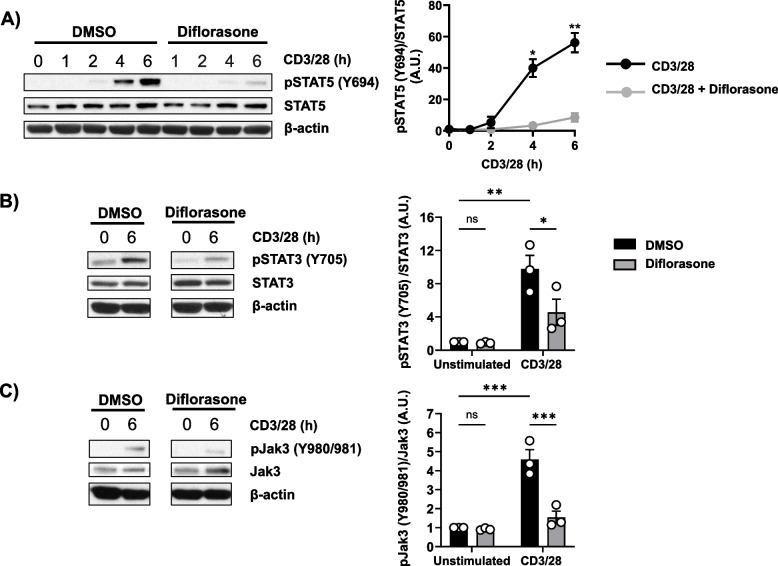
Impaired cytokine signalling in diflorasone-treated human T cells. Freshly isolated peripheral blood T cells were pretreated with 1µM diflorasone for 2 h and subsequently stimulated with CD3/28-coated beads. The phosphorylation levels and the expression of STAT5 (**A**), STAT3 (**B**), and Jak3 (**C**) were assessed in cell lysates using phospho-specific and total protein antibodies. β-actin was used as a loading control. A representative immunoblot is shown on the left side (4 donors were assayed in A and 3 donors in B and C). Bands in (**A**), (**B**), and (**C**) were quantified using ImageJ software by normalizing the signal of the phosphorylated molecule to the signal of the total target protein. Graphs on the right side show the phosphorylation levels of the indicated molecules as arbitrary units ± SEM of 3–4 experiments (1 donor was used in each experiment). Statistical analyses were performed using two-way ANOVA followed by Dunnett’s (STAT5) or Šídák’s (STAT3 and Jak3) post hoc test (*p ≤ 0.05, **p ≤ 0.01, and ***p ≤ 0.001)

Cytokines activate a variety of intracellular signalling cascades leading to gene transcription, which in turn regulates clonal expansion and differentiation of the activated T cells [33]. The Jak/STAT pathway is a well-known cytokine-mediated signalling cascade [34]. We therefore evaluated whether diflorasone has an effect on cytokine signalling by investigating Jak/STAT activation. We focused our analyses on Jak3, STAT3, and STAT5, which among others, are downstream of the IL-2R. We found that the phosphorylation of Jak3, STAT3, and STAT5 was either abrogated or strongly suppressed upon diflorasone treatment (Fig. 3), which is consistent with the hypothesis that diflorasone may interfere with cytokine signalling, particularly in the context of IL-2. On the other hand, we did not detect any phospho signal for Jak1 and Jak2 (data not shown).

**Fig. 4:**
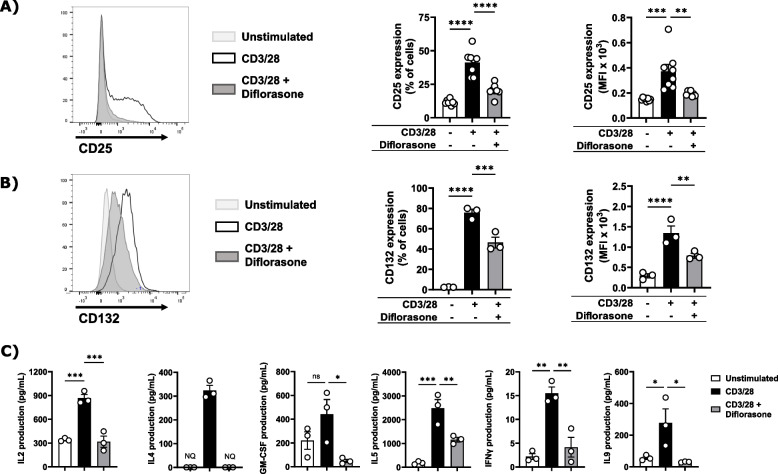
Diflorasone suppresses the upregulation of cytokine receptors and cytokine production in activated T cells. Diflorasone-treated human T cells were activated with CD3/28 antibodies for 24 h and the expression levels of CD25 (**A**) and CD132 (**B**) were assessed by flow cytometry. A representative histogram is shown on the left side. Graphs on the right side depict analyses of the expression of CD25 and CD132 presented as percentages of positive cells or as mean fluorescence intensity (MFI) ± SEM from 3–8 independent experiments (1 donor was used in each experiment). **C** Diflorasone-treated human T cells were activated with CD3/28 antibodies for 6 h. Subsequently, supernatants were collected and the production of IL2, GM-CSF, IL4, IL9, IL5, and IFNγ was measured. Data represent the concentration of cytokines expressed as pg/ml ± SEM from 3 independent experiments (1 donor was used in each experiment). Significance was determined using one-way ANOVA followed by Dunnett’s post hoc test (****p < 0.0001, ***p < 0.001, **p < 0.01, and *p < 0.05, ns = not statistically significant, NQ = not detectable)

Diflorasone could act at different levels to block IL-2 signalling. First, diflorasone can inhibit cytokine production as reported for other GCs. Second, diflorasone may affect the expression of the IL-2R and, third, it can directly inhibit the activation of signalling molecules downstream of the IL-2R, as previously reported in T-cell blasts [18]. To shed light onto how diflorasone inhibits IL-2 signalling in primary human T cells, we initially measured the expression of the high affinity IL-2 receptor (CD25), which is important to drive T-cell proliferation [35], and the expression of the common gamma chain (CD132), which is an essential signalling component of the IL-2R and many different interleukin receptors [36]. As shown in Fig. 4A-B, diflorasone treatment led to a marked suppression in the upregulation of both CD25 and CD132 in T cells stimulated with CD3/CD28 antibodies for 24 h.

In addition, we measured cytokine production. To this aim, T cells were treated either with diflorasone or with a control vehicle for 2 h and subsequently stimulated using CD3/CD28 antibodies for 6 h. Supernatants were collected and assayed for cytokine production using a MACSPlex assay. We found that diflorasone strongly inhibited the release of IL-2 and other cytokines in the culture supernatant of CD3/CD28-stimulated T cells (Fig. 4C).

Taken together, these results suggest that diflorasone rapidly impairs cytokine signalling and particularly the IL-2/IL-2R axis by suppressing IL-2 production and the expression of the IL-2R in primary human T cells.

### A common mechanism of action mediates the immunosuppressive effect of GCs in T cells

The data shown above suggest that diflorasone may have a partially distinct mechanism of action compared to the widely employed dexamethasone and prednisolone. Indeed, data published about two decades ago suggested that dexamethasone and prednisolone rapidly inhibit Lck activation and hence proximal TCR signaling [21, 22], whereas our data shown in Fig. 2A-D indicate that diflorasone does not. The published data also led to the conclusion that GCs have a novel non-genomic mechanism of action, which quickly suppresses TCR signaling upstream of and independently on the modulation of gene transcription [7]. To shed light on this issue, we performed experiments to reexamine this hypothesis.

Similar to the results shown in Fig. 1, the CD3/CD28-mediated proliferation of human peripheral blood T cells incubated with the well-known GCs dexamethasone and prednisolone was also strongly inhibited, thus confirming the immunosuppressive effects of the two GCs also under our experimental conditions (Figure S2). The block in proliferation was not due to an increase in apoptosis (Figure S2).

**Fig. 5:**
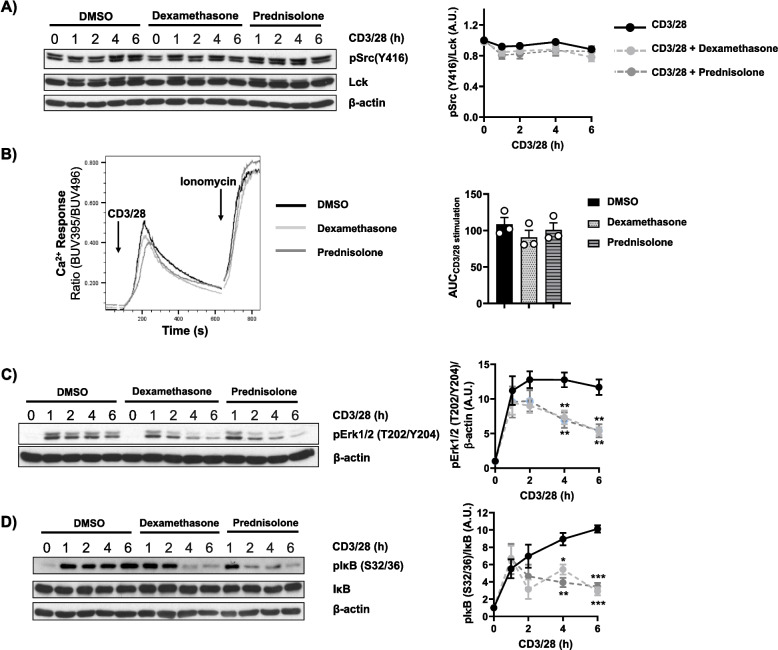
Dexamethasone and prednisolone do not inhibit Lck function and TCR-mediated signalling but inhibits sustained Erk1/2 and IκB phosphorylation in CD3/CD28-activated human T cells. Freshly isolated T cells were treated with 1µM dexamethasone or prednisolone and subsequently stimulated with CD3/28-coated beads for the indicated time points. Phosphorylation levels of Lck on Y394 (which were detected using the phospho-Src antibody recognizing the highly conserved across Src-family kinases Y416) and Lck expression (**A**), phosphorylation of Erk1/2 (T202/Y204) (**C**), phosphorylation of IκB (S32/36) and IκB expression (**D**) were assessed by immunoblotting. β-actin was used as a loading control. A representative immunoblot is presented on the left side (*n* = 4–6 donors). Quantification of the immunoblots was performed using the ImageJ software by normalizing the signal of the phosphorylated molecule to the signal of the total target protein or β-actin. Graphs on the right side show the phosphorylation levels of the indicated molecules as arbitrary units ± SEM from 4–6 experiments (1 donor was used in each experiment). Statistical analyses were performed using two-way ANOVA followed by Šídák’s post hoc test (*p ≤ 0.05 and **p ≤ 0.01, *** *p* ≤ 0.001, and **** *p* ≤ 0.0001). **B** Ca^2+^ influx was evaluated by flow cytometry upon CD3/28 stimulation of human T cells treated with dexamethasone and prednisolone. Ionomycin was employed to demonstrate equal loading with Indo-1. A representative experiment is displayed (*n* = 3 donors). Quantification of the Area Under the Curve (AUC) upon CD3/28 stimulation area is presented on the right side

**Fig. 6:**
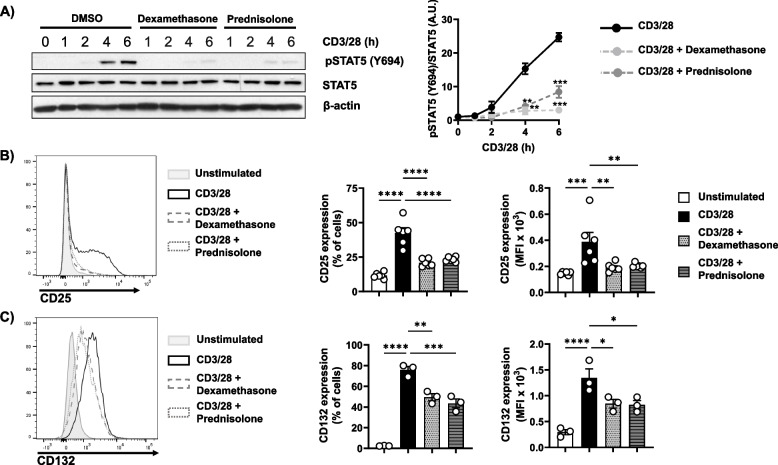
Dexamethasone and prednisolone suppress STAT5 phosphorylation and the upregulation of CD25 and CD132 in activated T cells. Freshly isolated T cells were treated with 1µM dexamethasone or prednisolone and subsequently stimulated with CD3/28-coated beads for the indicated time points. **A** Phosphorylation levels of STAT5 (Y694) or STAT5 expression were assessed in cell lysates by immunoblotting, with β-actin serving as a loading control. A representative immunoblot is presented on the left side of the figure (*n* = 4 donors). Graphs on the right side show the phosphorylation levels of STAT5 as arbitrary units ± SEM from 4 experiments (1 donor was used in each experiment). Band quantification was performed using ImageJ software and values from phosphorylated STAT5 were normalized to the signal of STAT5. Statistical analyses were performed using two-way ANOVA followed by Dunnett’s post hoc test (*p ≤ 0.05, **p ≤ 0.01, and ****p ≤ 0.0001). **B-C** The expressions of CD25 and CD132 were analyzed 24 h after stimulation by flow cytometry. Representative histograms are shown on the left side. Graphs on the right side depict quantification analyses of the expression of CD25 and CD132 presented as percentages of positive cells or as mean fluorescent intensity (MFI) ± SEM from 3 independent experiments (1 donor was used in each experiment). p-values were calculated using one-way ANOVA followed by Dunnett’s post hoc test (**p < 0.01, ***p < 0.001 and ****p < 0.0001 versus CD3/28 control)

To better understand how dexamethasone and prednisolone exert their immunosuppressive function, we performed experiments similar to those described above for diflorasone. We first measured the phosphorylation and the expression levels of Lck upon treatment with dexamethasone and prednisolone. To our surprise, we did not observe the inhibitory effects of both GCs on the phosphorylation of Lck (Fig. 5A), which was described in previous studies [21, 22]. To corroborate this observation, we assessed Lck phosphorylation by intracellular staining and extended the analysis to different T-cell subsets to rule out subset-specific effects. As shown in Figure S3, neither diflorasone, dexamethasone, nor prednisolone affected Lck activation in CD4⁺ or CD8⁺ T cells, whether naïve or memory. Similarly, Lck expression was not affected by dexamethasone and prednisolone (Fig. 5A). Accordingly, the TCR-mediated Ca^2+^ influx was grossly normal upon treatment with the two well-known GCs (Fig. 5B-C). Taken together, the data shown above indicate that, similar to diflorasone, dexamethasone and prednisolone do not suppress T-cell proliferation by impairing TCR signaling under our experimental conditions. Of note, dexamethasone and prednisolone inhibited Erk1/2 phosphorylation at later (2–6 h), but not at early stages (1 h) of T-cell activation again in a similar fashion to the effect of diflorasone (Fig. 5D-E). This observation suggests that dexamethasone and prednisolone may primarily affect cytokine-mediated signaling rather than TCR-induced signaling, similar to the effects observed with diflorasone. We next tested whether dexamethasone and prednisolone inhibit T-cell proliferation by affecting the IL-2 signaling axis. To this aim, we analyzed (i) the levels of STAT5 phosphorylation (Fig. 6A) and (ii) the expression of the cytokine receptors CD25 (Fig. 6B) and CD132 (Fig. 6C). Similar to the effect of diflorasone, we found that both dexamethasone and prednisolone also inhibited the induction of STAT5 phosphorylation and the upregulation of CD25 and CD132 in TCR-stimulated cells.

**Fig. 7:**
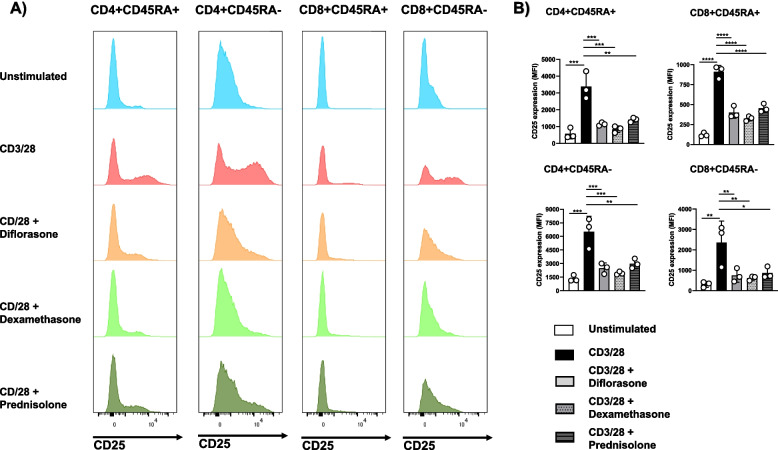
Treatment with GCs inhibits CD25 upregulation on different T-cell subsets. Freshly isolated T cells were treated with 1 µM diflorasone, dexamethasone, or prednisolone for 2 h and subsequently stimulated with CD3/28-coated beads for 24 h. After activation, T cells were stained with CD4, CD8, CD25, and CD45RA antibodies and analyzed by flow cytometry. **A** The levels of CD25 were assessed upon gaiting on different T-cell subsets as indicated in the histograms. Data from one representative donor are shown. **B** Graphs on the right side depict quantification analyses from 3 donors tested in 3 independent experiments. *p*-values were calculated using two-way ANOVA followed by Dunnett’s post hoc test (*p < 0.05, **p < 0.01, ***p < 0.001, and ****p < 0.0001 versus CD3/28 control)

The effects of GCs on T-cell functions were striking, leading us to hypothesize that all T-cell subsets are similarly affected. To test this assumption, we analyzed the impact of GCs on CD25 expression in T-cell subpopulations using flow cytometry. Cells were stained for CD4, CD8, and CD45RA to distinguish between memory and naïve T cells. As shown in Fig. 7, CD25 upregulation following CD3/CD28 stimulation was, as expected, more pronounced in both CD4⁺ and CD8⁺ memory T cells compared to their naïve counterparts. Importantly, GCs suppressed CD25 expression across all T-cell subsets to a similar extent (Fig. 7), indicating that the inhibitory effects of GCs are not subset-specific.

**Fig. 8:**
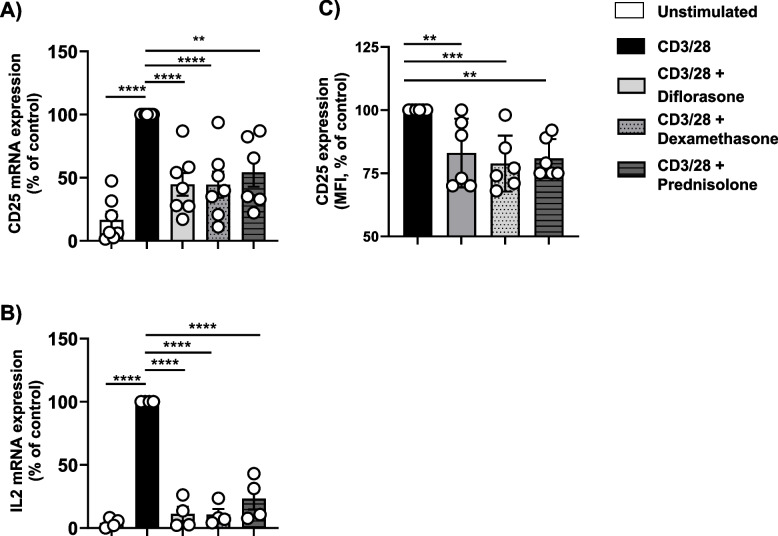
Treatment with GCs inhibits CD25 expression. Freshly isolated T cells were treated with 1 µM diflorasone, dexamethasone, or prednisolone for 2 h and subsequently stimulated with CD3/28-coated beads for 6 h. The levels of CD25 mRNA (**A**) and IL-2 (**B**) were assessed by RT-PCR. The graphs shows CD25 or IL-2 expression from four different donors represented as percentages ± SEM compared to the CD3/CD28-stimulated sample not treated with GCs. Each dot in (A) and (B) represent a donor. 1 or 2 donors were included in each experiment. (**C**) the graph depicts analysis of CD25 expression by flow cytometry on CD3/CD28-stimulated T cells which were left untreated or treated with GCs. Data are presented as MFI normalized to untreated cells ± SEM from 3 independent experiments (2 donors were included in each experiment). p-values were calculated using two-way ANOVA followed by Dunnett’s post hoc test (**p < 0.01, ***p < 0.001, and ****p < 0.0001 versus CD3/28 control)

The data presented above suggest that GCs may suppress interleukin signaling by downregulating the IL-2R and/or IL-2 production. However, while CD25 and CD132 expression was assessed 24 h after CD3/CD28 stimulation, signaling studies were conducted at much earlier time points (4–6 h). Therefore, it remains unclear whether GCs inhibit interleukin signaling primarily by reducing IL-2R or IL-2 expression, as suggested by the current data and the literature, or whether they interfere with downstream signal transmission. To address this, we examined mRNA levels of IL-2 and CD25 expression at both the mRNA and protein levels 6 h after stimulation to align with the signaling data. We found that all tested GCs rapidly suppressed IL-2 mRNA (corroborating the data on the protein levels shown in Fig. 4C) and reduced CD25 mRNA transcription (Fig. 8A, B). We next assessed CD25 protein expression by flow cytometry. As expected, CD25 surface expression on resting T cells was minimal, and CD3/CD28 stimulation did not induce a significant upregulation at the 6-h time point (not shown), unlike the more robust upregulation observed at 24 h. Nevertheless, all GCs used in our study decreased CD25 expression (Fig. 8C). Similarly, we observed that GCs also modestly reduced the surface expression of CD132 (Figure S4). Although the very low expression of CD25 and CD132 detected by flow cytometry at 6 h post-stimulation makes it difficult to draw a clear conclusion, the analysis of CD25 mRNA suggests that GC treatment might rapidly prevent the upregulation of the IL-2R.

**Fig. 9:**
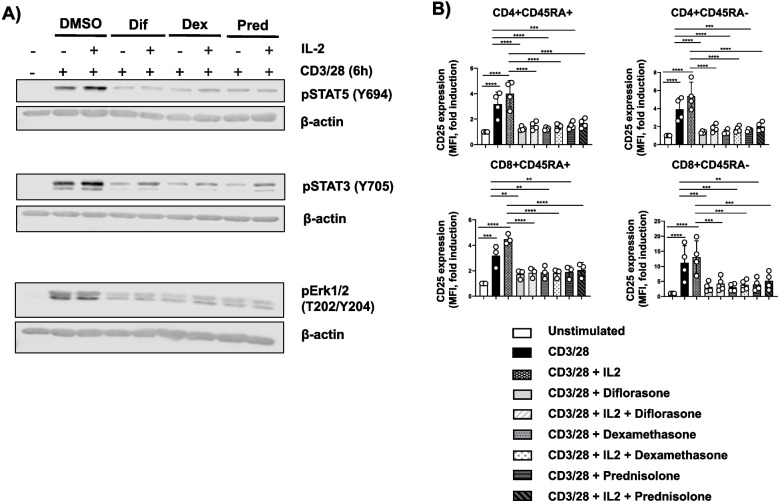
Exogenous IL-2 does not rescue Jak/STAT signaling and CD25 expression. Freshly isolated T cells were treated for 2 h with 1 µM diflorasone, dexamethasone, or prednisolone, followed by 6-h stimulation with CD3/CD28-coated beads in the presence or absence of recombinant IL-2. **A** Phosphorylation levels of STAT5 (Y694), STAT3 (Y705), and Erk1/2 (T202/Y204) were measured in cell lysates by immunoblotting, with β-actin used as a loading control. A representative immunoblot is shown, with data from 3 donors across 3 independent experiments. **B** The graphs display CD25 expression in various T-cell subsets (CD4, CD8, and CD45RA) after staining. Data from 3–4 donors in 3–4 independent experiments are shown as individual points (1 donor was used in each experiment). p-values were calculated using two-way ANOVA followed by Dunnett’s post hoc test (**p < 0.01, ***p < 0.001, and ****p < 0.0001)

We next investigated whether the defective signaling was primarily due to reduced IL-2 production. To this end, we added exogenous IL-2 to CD3/CD28-stimulated cells with or without GC treatment. Exogenous IL-2 failed to restore phosphorylation of STAT5, STAT3, and Erk1/2 (Fig. 9A). Similarly, it did not rescue CD25 expression in any of the T-cell subsets treated with GCs (Fig. 9B), nor did it reverse the GC-mediated suppression of T-cell proliferation observed with diflorasone treatment (Figure S5). These findings indicate that impaired IL-2 production alone does not fully account for the functional defects induced by GCs.

**Fig. 10:**
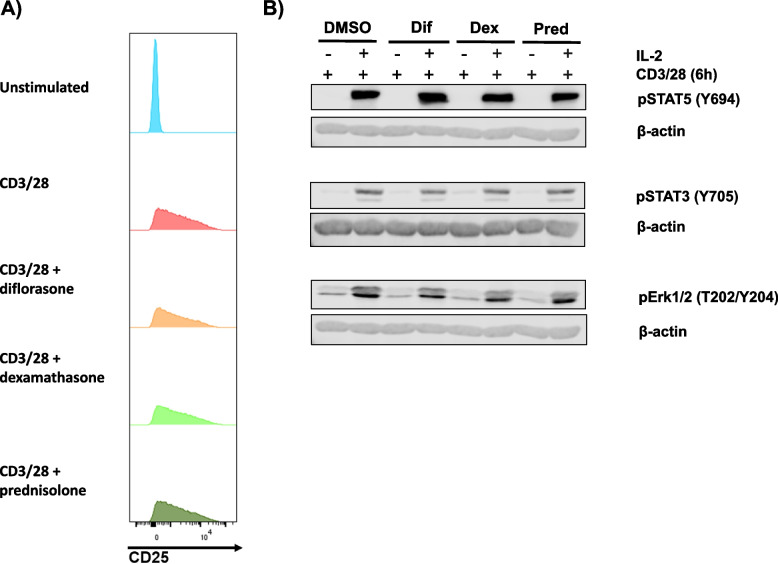
Jak/STAT signaling is not affected in T-cell blasts treated with GCs. PBMCs were stimulated for 48 h with CD3/CD28 antibodies immobilized on cell culture dishes. After activation, cells were rested for 24 h and subsequently incubated with GCs (1 µM each) for 2 h. **A** CD25 expression was measured by flow cytometry after GC-treatment. Data from one representative donor are shown. **B** T-cell blasts were stimulated with recombinant IL-2 for 20 min and phosphorylation levels of STAT5 (Y694), STAT3 (Y705), and Erk1/2 (T202/Y204) were measured in cell lysates by immunoblotting, with β-actin used as a loading control. Data from one representative donor are shown. 1 donor in each independent experiments was tested (*n* = 3 experiments)

To test the effects of GCs on the IL-2R signaling machinery, we generated T-cell blasts in the absence of GCs to ensure robust CD25 expression. T-cell blasts were subsequently preincubated with GCs for 2 h and then stimulated with IL-2. Under these conditions, GCs did not significantly affect CD25 expression (Fig. 10A) or the phosphorylation of STAT5, STAT3, Erk1/2, Jak3, or Jak1 (Fig. 10B and data not shown). Collectively, these data indicate that GCs do not directly inhibit IL-2 signaling when IL-2R expression is already established.

In summary, our findings suggest that GCs exert their immunosuppressive effects by rapidly impairing the expression of cytokines (e.g., IL-2) and cytokine receptors (e.g., CD25, CD132), thereby disrupting Jak/STAT signaling in primary human T cells. Conversely, GCs do not appear to suppress T-cell receptor (TCR) signaling at least at the proximal level. The rapid disruption of the mitogenic IL-2/IL-2R signaling axis likely impairs early T-cell activation and contributes to the inhibition of proliferation and effector function. We propose that this mechanism is a key contributor to the therapeutic efficacy of GCs.

## Discussion

GCs are important therapeutic agents to treat a variety of immune-related diseases. Over the past years several studies have been conducted to understand how these important immunosuppressants inhibit T-cell functions. Despite these attempts and many years of research, it is still unclear how GCs function. Indeed, a variety of both genomic and non-genomic immunosuppressive mechanisms of action have been described, which are reviewed in [2, 5, 7, 8, 11]. These different effects of GCs in T cells are likely due to the different experimental conditions used in the studies, including cell type (e.g. primary T cells vs. T-cell lines or T-cell blasts), concentration of the GCs, etc. Additionally, also the pleiotropic effects of GCs, which may exert different effects depending not only on the cell type but also on the incubation time, might have played a role in the observed discrepancies. Due to the therapeutic importance of GCs and the reported inconsistencies regarding their mechanisms of action, we thought that it is important to look closer at this issue.

In our work, we focused our investigations on the effects of GCs on TCR and cytokine signaling, in particular IL-2. Both TCR and IL-2R signaling regulate T-cell activation and proliferation, although at different phases. To discriminate *bona fide* between TCR and IL-2R signaling in our experimental setting, we assessed the activation kinetics of Zap70/LAT and STAT5, which regulate TCR and IL-2R signaling, respectively. As suggested by the activation kinetics of Zap70 and LAT (Fig. 2B-C), TCR signaling is rapidly induced 1–2 h after TCR/CD28 stimulation but declines during the subsequent 4–6 h. On the other hand, STAT5 phosphorylation becomes evident only 4–6 h after stimulation (Fig. 3A). Our data indicate that the most striking effect of GCs is the suppression of STAT5 phosphorylation (and also Jak3 and STAT3) 4–6 h upon TCR/CD28 stimulation. Conversely, we did not observe any major effect of GCs on TCR-mediated signaling events 1–2 h upon TCR/CD28 stimulation. When we looked at signaling pathways, such as MAPK and NF-κB, which are downstream of both TCR and cytokine receptors, we found that GCs did not inhibit the induction of Erk1/2 and IκB phosphorylation 1–2 h after stimulation, which is TCR-mediated. Rather, GCs impaired their sustained activation with a major effect detectable 4–6 h after stimulation, which mirrored the suppressing effect of GCs on STAT5 phosphorylation. Based on these observations, we conclude that GCs primarily and rapidly inhibit IL-2 (and more generally cytokine), but not TCR signaling. Thus, our study identifies one of the earliest levels of GC action in primary human T cells. We show for the first time that GCs rapidly inhibit the IL-2/IL-2R signaling axis in primary human T cells and we further corroborate previous observations obtained using T-cell blasts [18]. As IL-2 signaling is of pivotal importance for T-cell expansion and survival, we believe that this mechanism substantially contributes to the potent immunosuppressive effect of GCs in T cells.

The question that remains open is how GCs influence IL-2 signaling. In this regard, we propose three possible mechanisms. First, GCs may impair IL-2 signaling by suppressing IL-2 production. Indeed, we observed a marked reduction in IL-2 expression 6 h after stimulation. However, adding exogenous IL-2 does not rescue the GC-induced defects in Jak/STAT signaling, CD25 upregulation, or proliferation (at least for diflorasone). This indicates that reduced IL-2 production alone is not sufficient to explain the observed signaling defects. Second, GCs may inhibit components of the signaling machinery downstream of the IL-2R. However, IL-2 signaling remains intact in T-cell blasts pre-incubated with GCs, suggesting that GCs do not directly block downstream signal transduction. Third, GCs may impair IL-2 signaling by preventing IL-2R expression. We observed reduced expression of both CD25 and CD132 24 h after CD3/CD28 stimulation in GC-treated cells. However, this time point differs from that at which IL-2 production and Jak/STAT signaling were initially impaired (6 h after CD3/CD28 stimulation). To clarify this, we analyzed CD25 expression at earlier time points at both the mRNA and protein level. While CD25 expression is generally low at early activation stages, we detected a slight but consistent reduction in both CD25 mRNA and surface expression upon GC treatment.

Collectively, these results suggest that GCs might inhibit T-cell activation primarily by impairing the early upregulation of CD25. This may involve the glucocorticoid receptor (GR), which is known to interfere with the cooperative activity of NFAT and AP-1 in promoting IL-2 transcription, as shown in Jurkat T cells [37]. Given that CD25 transcription is also regulated by NFAT and AP-1 through adjacent binding sites in its promoter [38, 39], it is plausible that GCs suppress CD25 expression through a similar mechanism. When comparing the effects of GCs on IL-2 and CD25 mRNA levels, we found that IL-2 transcription was more strongly inhibited. This observation aligns with previous reports showing that GCs potently suppress IL-2 mRNA expression, while having a more modest inhibitory effect on CD25 expression [40, 41]. These studies further suggested that GCs regulate CD25 expression at both transcriptional and post-transcriptional levels.

As mentioned above, it is well established that GCs can directly suppress gene transcription by binding to the glucocorticoid responsive elements in the promoter region of target genes. However, it has been shown that GCs can suppress gene transcription also indirectly by upregulating the expression of proteins inhibiting transcription, such as the glucocorticoid-induced leucine zipper (GILZ) [11]. One interesting model postulates that GCs inhibit T-cell proliferation by inducing the rapid transcription of the immunosuppressive gene GILZ, which in turn inhibits NF-κB [42]. GILZ may also inhibit AP-1 transcriptional activity by binding rat sarcoma virus protein (Ras) and Raf-1 and downregulating Ras signaling [43, 44]. Interestingly, suppression of GILZ expression prevented the antiproliferative effect of dexamethasone on Concanavalin A -mediated T-cell proliferation [44]. Based upon these observations, it is plausible that GCs suppress IL-2 and IL-2R expression not only directly, but also indirectly by impairing the activation of the transcription factors AP1 and NF-κB via GILZ. This hypothesis is supported by our data showing that GC treatment reduces sustained Erk1/2 and IκB phosphorylation. Impaired activation of AP-1 and NF-κB could, in turn, lead to diminished IL-2 production and reduced IL-2R expression, ultimately resulting in attenuated Jak/STAT signaling and T-cell activation. We cannot exclude the possibility that GCs inhibit sustained Erk1/2 and IκB phosphorylation by interfering with TCR signaling downstream of LAT phosphorylation at later stages of T-cell activation. While our study did not specifically investigate the Notch signaling pathway, it is plausible that GCs may influence it indirectly. For example, Zanotti et al. demonstrated that GCs can inhibit Notch signaling in osteoblasts [45]. Whether a similar mechanism occurs in T cells, potentially contributing to the modulation of TCR activity, remains an interesting possibility that warrants further investigation. In the future, the effect of GCs on T-cell proliferation should be assessed not only by the standard DNA-synthesis [^3^H]-thymidine incorporation assay, but also by additional methods such as Ki-67 staining and CFSE dilution. 

## Conclusions

We have shown here that the rapid suppression of the IL-2/IL-2R signaling axis, but not of proximal TCR signaling, is one of the earliest detectable immunosuppressive effects of GCs during T-cell activation. Thus, our study has highlighted a clear mechanism of action of GCs in T cells contributing to their therapeutic efficacy.

## Supplementary Information

Supplementary Material 1.

## Data Availability

No datasets were generated or analysed during the current study.

## Abbreviations

7 AAD: 7-Aminoactinomycin D
APC: Allophycocyanin
APCs: Antigen-presenting cells
DMSO: Dimethyl sulfoxide
Erk1/2: Extracellular signal-regulated kinase 1/2
FBS: Fetal bovine serum
GCs: Glucocorticoids
GCR: Glucocorticoid receptor
GILZ: Glucocorticoid-induced leucine zipper
IκB: Inhibitor of kappa B
IC50: Half-maximal inhibitory concentration
IL-2R: Interleukin-2 receptor
Itk: Interleukin-2-inducible T-cell kinase
Jak: Janus kinase
LAT: Linker for activation of T cells
Lck: Lymphocyte-specific protein tyrosine kinase
MAPK: Mitogen-activated protein kinase
NF-κB: Nuclear factor kappa B
PBMCs: Peripheral blood mononuclear cells
PBS: Phosphate-buffered saline
PD-1: Programmed cell death protein 1
PE: Phycoerythrin
PMA: Phorbol 12-myristate 13-acetate
Ras: Rat sarcoma virus protein
RT: Room temperature
SDS-PAGE: Sodium dodecyl sulfate‒polyacrylamide gel electrophoresis
STAT: Signal transducer and activator of transcription
TBS: Tris-buffered saline
TdR: Thymidine
TCR: T-cell receptor
Zap70: Zeta-chain-associated protein kinase 70

## References

[1] Alexander SP, Cidlowski JA, Kelly E, Marrion NV, Peters JA, Faccenda E, et al. The Concise Guide to Pharmacology 2017/18: Nuclear hormone receptors. Br J Pharmacol. 2017;174 Suppl 1(Suppl Suppl 1):S208–24. 29055032 10.1111/bph.13880 PMC5650662

[2] Cain DW, Cidlowski JA. Immune regulation by glucocorticoids. Nat Rev Immunol. 2017;17(4):233–47. 28192415 10.1038/nri.2017.1 PMC9761406

[3] Rhen T, Cidlowski JA. Antiinflammatory action of glucocorticoids–new mechanisms for old drugs. N Engl J Med. 2005;353(16):1711–23. 16236742 10.1056/NEJMra050541

[4] Reichardt SD, Amouret A, Muzzi C, Vettorazzi S, Tuckermann JP, Luhder F, et al. The Role of Glucocorticoids in Inflammatory Diseases. Cells. 2021;10(11):2921. 34831143 10.3390/cells10112921 PMC8616489

[5] Taves MD, Ashwell JD. Glucocorticoids in T cell development, differentiation and function. Nat Rev Immunol. 2021;21(4):233–43. 33149283 10.1038/s41577-020-00464-0

[6] Borin C, Pieters T, Serafin V, Ntziachristos P. Emerging Epigenetic and Posttranslational Mechanisms Controlling Resistance to Glucocorticoids in Acute Lymphoblastic Leukemia. Hemasphere. 2023;7(7):e916. 37359189 10.1097/HS9.0000000000000916 PMC10289758

[7] Lowenberg M, Verhaar AP, van den Brink GR, Hommes DW. Glucocorticoid signaling: a nongenomic mechanism for T-cell immunosuppression. Trends Mol Med. 2007;13(4):158–63. 17293163 10.1016/j.molmed.2007.02.001

[8] Jia WY, Zhang JJ. Effects of glucocorticoids on leukocytes: Genomic and non-genomic mechanisms. World J Clin Cases. 2022;10(21):7187–94. 36158016 10.12998/wjcc.v10.i21.7187 PMC9353929

[9] Panettieri RA, Schaafsma D, Amrani Y, Koziol-White C, Ostrom R, Tliba O. Non-genomic Effects of Glucocorticoids: An Updated View. Trends Pharmacol Sci. 2019;40(1):38–49. 30497693 10.1016/j.tips.2018.11.002 PMC7106476

[10] Boldizsar F, Talaber G, Szabo M, Bartis D, Palinkas L, Nemeth P, et al. Emerging pathways of non-genomic glucocorticoid (GC) signalling in T cells. Immunobiology. 2010;215(7):521–6. 19906460 10.1016/j.imbio.2009.10.003

[11] Cannarile L, Delfino DV, Adorisio S, Riccardi C, Ayroldi E. Implicating the Role of GILZ in Glucocorticoid Modulation of T-Cell Activation. Front Immunol. 2019;10:1823. 31440237 10.3389/fimmu.2019.01823 PMC6693389

[12] Shimba A, Ikuta K. Control of immunity by glucocorticoids in health and disease. Semin Immunopathol. 2020;42(6):669–80. 33219395 10.1007/s00281-020-00827-8

[13] Zen M, Canova M, Campana C, Bettio S, Nalotto L, Rampudda M, et al. The kaleidoscope of glucorticoid effects on immune system. Autoimmun Rev. 2011;10(6):305–10. 21224015 10.1016/j.autrev.2010.11.009

[14] Randazzo B, Hirschberg T, Hirschberg H. Inhibition of the antigen activated T cell response by methylprednisolone is caused by inhibition of interleukin-2 (IL-2) production. Int J Immunopharmacol. 1984;6(5):419–23. 6334041 10.1016/0192-0561(84)90079-1

[15] Arya SK, Wong-Staal F, Gallo RC. Dexamethasone-mediated inhibition of human T cell growth factor and gamma-interferon messenger RNA. J Immunol. 1984;133(1):273–6. 6427338

[16] Goodwin JS, Atluru D, Sierakowski S, Lianos EA. Mechanism of action of glucocorticosteroids. Inhibition of T cell proliferation and interleukin 2 production by hydrocortisone is reversed by leukotriene B4. J Clin Invest. 1986;77(4):1244–50. 3007577 10.1172/JCI112427 PMC424468

[17] Lillehoj H, Shevach EM. A comparison of the effects of cyclosporin A, dexamethasone, and ouabain on the interleukin-2 cascade. J Immunopharmacol. 1985;7(3):267–84. 3932520 10.3109/08923978509026476

[18] Bianchi M, Meng C, Ivashkiv LB. Inhibition of IL-2-induced Jak-STAT signaling by glucocorticoids. Proc Natl Acad Sci U S A. 2000;97(17):9573–8. 10920190 10.1073/pnas.160099797 PMC16906

[19] Xing K, Gu B, Zhang P, Wu X. Dexamethasone enhances programmed cell death 1 (PD-1) expression during T cell activation: an insight into the optimum application of glucocorticoids in anti-cancer therapy. BMC Immunol. 2015;16:39. 26112261 10.1186/s12865-015-0103-2 PMC4480888

[20] Maeda N, Maruhashi T, Sugiura D, Shimizu K, Okazaki IM, Okazaki T. Glucocorticoids potentiate the inhibitory capacity of programmed cell death 1 by up-regulating its expression on T cells. J Biol Chem. 2019;294(52):19896–906. 31723031 10.1074/jbc.RA119.010379 PMC6937557

[21] Lowenberg M, Tuynman J, Bilderbeek J, Gaber T, Buttgereit F, van Deventer S, et al. Rapid immunosuppressive effects of glucocorticoids mediated through Lck and Fyn. Blood. 2005;106(5):1703–10. 15899916 10.1182/blood-2004-12-4790

[22] Lowenberg M, Verhaar AP, Bilderbeek J, Marle J, Buttgereit F, Peppelenbosch MP, et al. Glucocorticoids cause rapid dissociation of a T-cell-receptor-associated protein complex containing LCK and FYN. EMBO Rep. 2006;7(10):1023–9. 16888650 10.1038/sj.embor.7400775 PMC1618362

[23] Ghosh MC, Baatar D, Collins G, Carter A, Indig F, Biragyn A, et al. Dexamethasone augments CXCR4-mediated signaling in resting human T cells via the activation of the Src kinase Lck. Blood. 2009;113(3):575–84. 18840710 10.1182/blood-2008-04-151803 PMC2628365

[24] Muller N, Fischer HJ, Tischner D, van den Brandt J, Reichardt HM. Glucocorticoids induce effector T cell depolarization via ERM proteins, thereby impeding migration and APC conjugation. J Immunol. 2013;190(8):4360–70. 23475220 10.4049/jimmunol.1201520

[25] Bluefarb SM, Howard FM, Leibsohn E, Schlagel CA, Wexler L. Diflorasone diacetate: vasoconstrictor activity and clinical efficacy of a new topical corticosteroid. J Int Med Res. 1976;4(6):454–61. 800385 10.1177/030006057600400613

[26] Jegasothy BV. Clobetasol propionate ointment 0.05% versus diflorasone diacetate ointment 0.05% in moderate to severe psoriasis. Int J Dermatol. 1990;29(10):729–30. 2269571 10.1111/j.1365-4362.1990.tb03781.x

[27] Shupack JL, Jondreau L, Kenny C, Stiller MJ. Diflorasone diacetate ointment 0.05% versus betamethasone dipropionate ointment 0.05% in moderate-severe plaque-type psoriasis. Dermatology. 1993;186(2):129–32. 8428041 10.1159/000247323

[28] Krueger GG, O’Reilly MA, Weidner M, Dromgoole SH, Killey FP. Comparative efficacy of once-daily flurandrenolide tape versus twice-daily diflorasone diacetate ointment in the treatment of psoriasis. J Am Acad Dermatol. 1998;38(2 Pt 1):186–90. 9486672 10.1016/s0190-9622(98)70238-5

[29] Zareifi DS, Chaliotis O, Chala N, Meimetis N, Sofotasiou M, Zeakis K, et al. A network-based computational and experimental framework for repurposing compounds toward the treatment of non-alcoholic fatty liver disease. Science. 2022;25(3):103890. 10.1016/j.isci.2022.103890 PMC8889147 35252807

[30] Warnecke N, Poltorak M, Kowtharapu BS, Arndt B, Stone JC, Schraven B, et al. TCR-mediated Erk activation does not depend on Sos and Grb2 in peripheral human T cells. EMBO Rep. 2012;13(4):386–91. 22344067 10.1038/embor.2012.17 PMC3321150

[31] Arndt B, Poltorak M, Kowtharapu BS, Reichardt P, Philipsen L, Lindquist JA, et al. Analysis of TCR activation kinetics in primary human T cells upon focal or soluble stimulation. J Immunol Methods. 2013;387(1–2):276–83. 23178863 10.1016/j.jim.2012.11.006

[32] Huang W, August A. The signaling symphony: T cell receptor tunes cytokine-mediated T cell differentiation. J Leukoc Biol. 2015;97(3):477–85. 25525115 10.1189/jlb.1RI0614-293R PMC4338847

[33] Dong C. Cytokine Regulation and Function in T Cells. Annu Rev Immunol. 2021;39:51–76. 33428453 10.1146/annurev-immunol-061020-053702

[34] Hu Q, Bian Q, Rong D, Wang L, Song J, Huang HS, et al. JAK/STAT pathway: Extracellular signals, diseases, immunity, and therapeutic regimens. Front Bioeng Biotechnol. 2023;11:1110765. 36911202 10.3389/fbioe.2023.1110765 PMC9995824

[35] Shatrova AN, Mityushova EV, Vassilieva IO, Aksenov ND, Zenin VV, Nikolsky NN, et al. Time-Dependent Regulation of IL-2R alpha-Chain (CD25) Expression by TCR Signal Strength and IL-2-Induced STAT5 Signaling in Activated Human Blood T Lymphocytes. PLoS ONE. 2016;11(12): e0167215. 27936140 10.1371/journal.pone.0167215 PMC5172478

[36] Rochman Y, Spolski R, Leonard WJ. New insights into the regulation of T cells by gamma(c) family cytokines. Nat Rev Immunol. 2009;9(7):480–90. 19543225 10.1038/nri2580 PMC2814538

[37] Vacca A, Felli MP, Farina AR, Martinotti S, Maroder M, Screpanti I, et al. Glucocorticoid receptor-mediated suppression of the interleukin 2 gene expression through impairment of the cooperativity between nuclear factor of activated T cells and AP-1 enhancer elements. J Exp Med. 1992;175(3):637–46. 1740658 10.1084/jem.175.3.637 PMC2119143

[38] Schuh K, Twardzik T, Kneitz B, Heyer J, Schimpl A, Serfling E. The interleukin 2 receptor alpha chain/CD25 promoter is a target for nuclear factor of activated T cells. J Exp Med. 1998;188(7):1369–73. 9763616 10.1084/jem.188.7.1369 PMC2212486

[39] Macian F, Lopez-Rodriguez C, Rao A. Partners in transcription: NFAT and AP-1. Oncogene. 2001;20(19):2476–89. 11402342 10.1038/sj.onc.1204386

[40] Reed JC, Abidi AH, Alpers JD, Hoover RG, Robb RJ, Nowell PC. Effect of cyclosporin A and dexamethasone on interleukin 2 receptor gene expression. J Immunol. 1986;137(1):150–4. 3011905

[41] Boumpas DT, Anastassiou ED, Older SA, Tsokos GC, Nelson DL, Balow JE. Dexamethasone inhibits human interleukin 2 but not interleukin 2 receptor gene expression in vitro at the level of nuclear transcription. J Clin Invest. 1991;87(5):1739–47. 2022743 10.1172/JCI115192 PMC295281

[42] Ayroldi E, Migliorati G, Bruscoli S, Marchetti C, Zollo O, Cannarile L, et al. Modulation of T-cell activation by the glucocorticoid-induced leucine zipper factor via inhibition of nuclear factor kappaB. Blood. 2001;98(3):743–53. 11468175 10.1182/blood.v98.3.743

[43] Ayroldi E, Zollo O, Macchiarulo A, Di Marco B, Marchetti C, Riccardi C. Glucocorticoid-induced leucine zipper inhibits the Raf-extracellular signal-regulated kinase pathway by binding to Raf-1. Mol Cell Biol. 2002;22(22):7929–41. 12391160 10.1128/MCB.22.22.7929-7941.2002 PMC134721

[44] Ayroldi E, Zollo O, Bastianelli A, Marchetti C, Agostini M, Di Virgilio R, et al. GILZ mediates the antiproliferative activity of glucocorticoids by negative regulation of Ras signaling. J Clin Invest. 2007;117(6):1605–15. 17492054 10.1172/JCI30724 PMC1865030

[45] Stefano, Zanotti Jungeun, Yu Suyash, Adhikari Ernesto, Canalis. Glucocorticoids inhibit notch target gene expression in osteoblasts Abstract. J Cell Biochem. 2018;119(7):6016–23. 10.1002/jcb.26798 PMC6830575 29575203

