# Supplementary material for: The immunosuppressive effect of glucocorticoids in human primary T cells is mainly mediated via a rapid inhibition of the IL-2/IL-2R signaling axis
Source: Cell Commun Signal. 2025 Jun 4;23:268. doi: 10.1186/s12964-025-02266-0 (PMC12139085; doi:10.1186/s12964-025-02266-0)
Supplement: Supplementary file 1 — Supplementary Material 1. [file 12964_2025_2266_MOESM1_ESM.pdf]

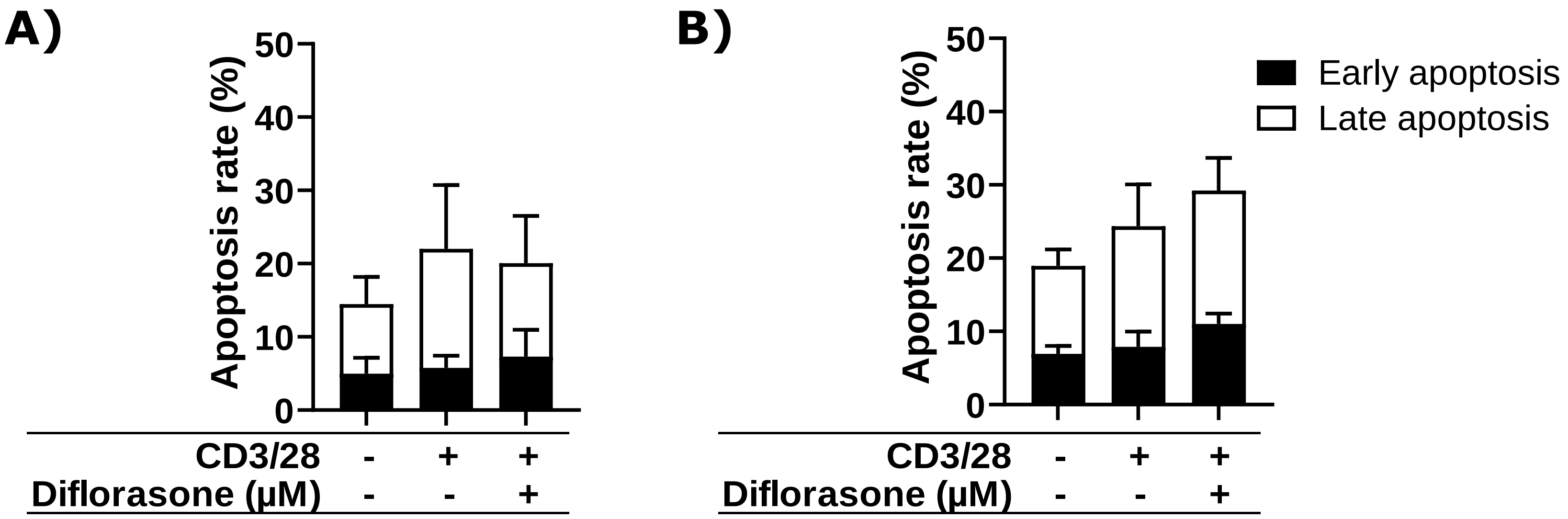

Figure S1. Diflorasone does not induce apoptosis in stimulated T cells. Freshly isolated human T cells were incubated with diflorasone and subsequently stimulated with CD3/CD28 antibodies. 24h **(A)** and 48h **(B)** after stimulation, T cells were stained with Annexin V/7AAD and assayed by flow cytometry. Early apoptotic cells were defined as Annexin $V^+/7AAD^-$, whereas late apoptotic cells were Annexin $V^+/7AAD^+$. Data represent the mean ± SEM of 3 independent experiments.

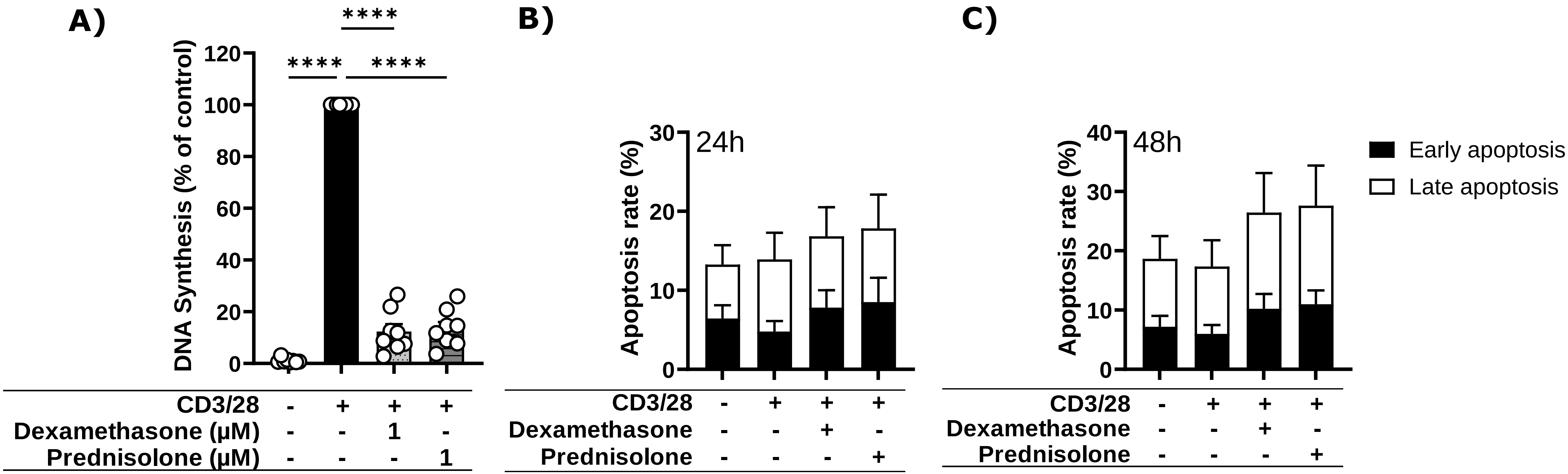

Figure S2. Dexamethasone and prednisolone inhibited the proliferation of stimulated human T cells without affecting apoptosis. Freshly isolated human T cells were treated with dexamethasone and prednisolone and subsequently stimulated with CD3/CD28 antibodies. **(A)** T-cell proliferation was determined by [^{3}H]-thymidine incorporation assay. Data are presented as the mean ± SEM of n = 4-8 independent experiments. T cells were stained after 24h **(B)** and 48h **(C)** with Annexin V/7AAD for a flow cytometer. Quantification of early and late apoptotic cells of 2 independent experiments. Early apoptotic cells were defined as Annexin V$^+$/7AAD$^-$, whereas late apoptotic cells were Annexin V$^+$/7AAD$^+$. Statistical analysis was performed with One-Way ANOVA with a post hoc test Dunnett's Multiple Comparison Analysis (* $p \leq 0.05$).

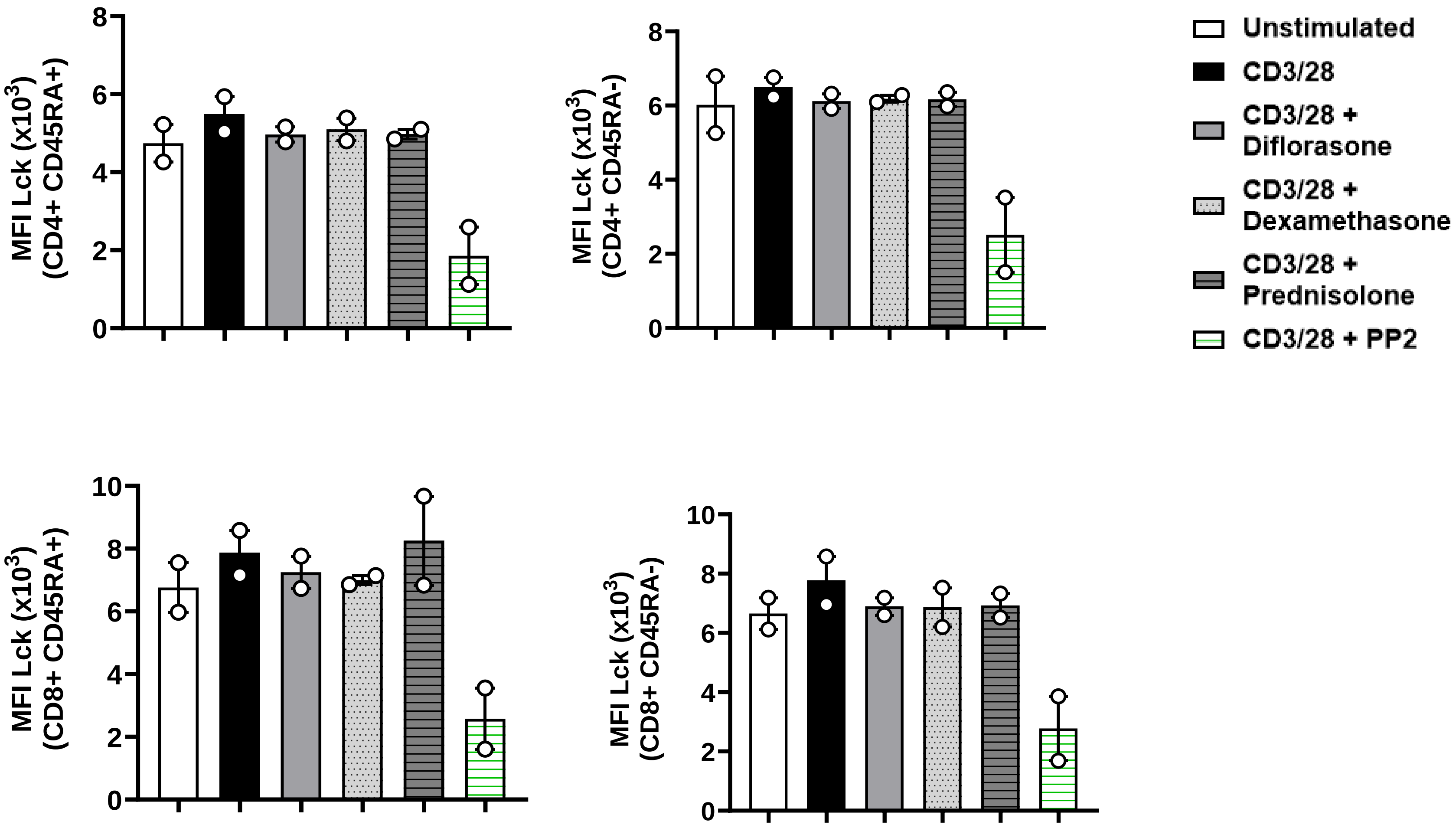

Figure S3. GCs do not affect Lck phosphorylation in different T-cell subsets. Freshly isolated human T cells were treated with diflorasone, dexamethasone, and prednisolone and subsequently stimulated with CD3/CD28 antibodies for 6 hours. After stimulation, T cells were stained with CD8, CD4, and CD45RA antibodies. Cells were then fixed for 20 minutes at room temperature in the dark. Subsequently, T cells were permeabilized and incubated with an anti-phospho Src (Y416) recognizing Lck phosphorylated on Y394 for 1 hour at 4°C in the dark. After staining, cells were washed with PBS and analyzed by flow cytometry. The levels of phosphorylated Lck were assessed upon gaiting on different T-cell subsets as indicated. Treatment with the Src inhibitor PP2 was used to decrease the levels of Y394 phosphorylation as control for antibody staining.

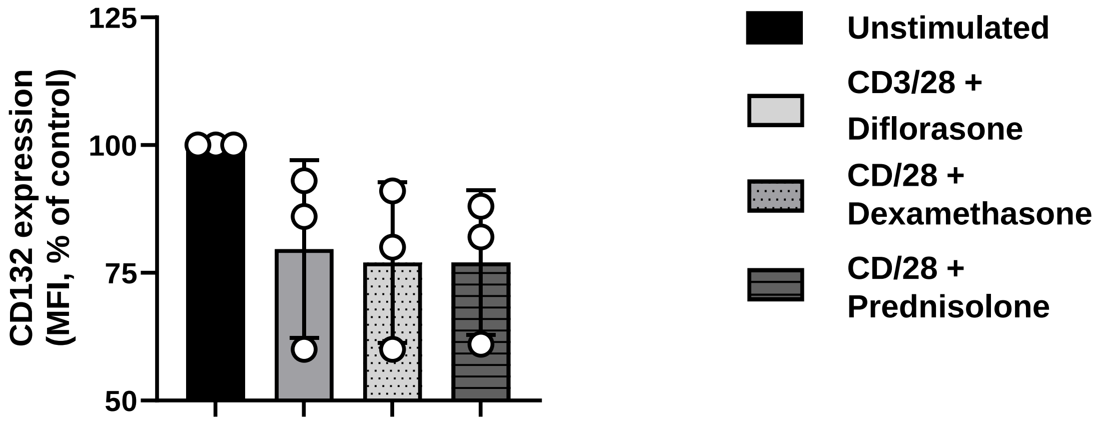

Figure S4. Treatment with GCs inhibits CD132 expression.
Freshly isolated T cells were treated with 1µM diflorasone, dexamethasone, or prednisolone for 2 hours and subsequently stimulated with CD3/28-coated beads for 6 hours. The levels of CD132 were assessed by flow cytometry. Data are presented as MFI normalized to untreated cells ± SEM from 3 independent experiments (1 donor was included in each experiment). Each dot represents 1 donor.

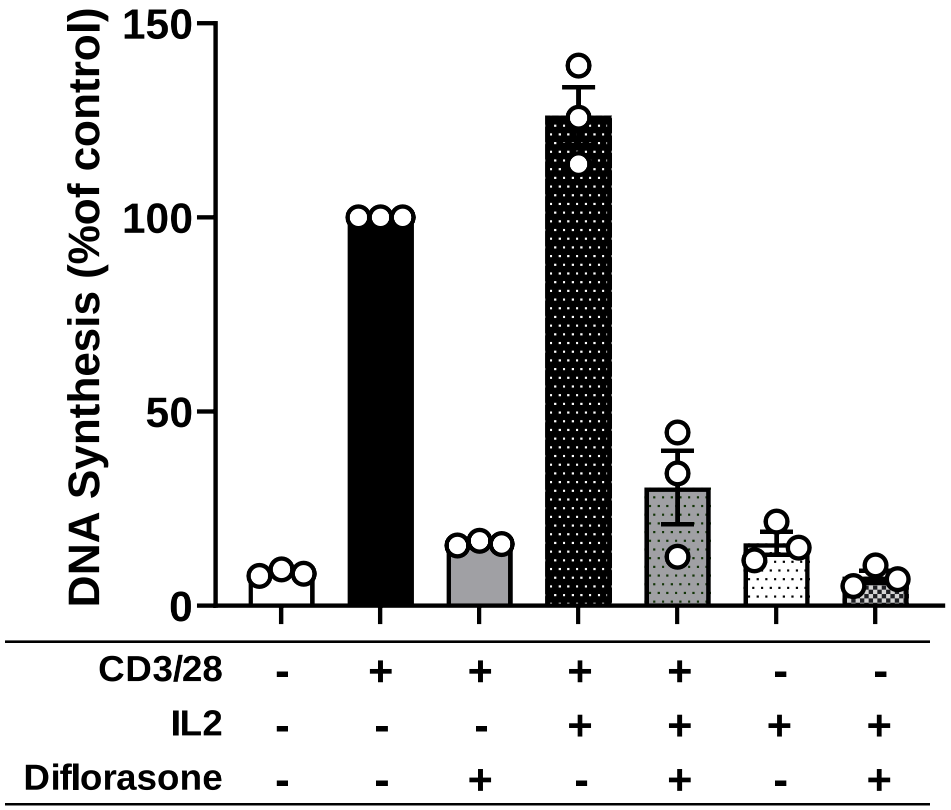

Figure S5. Exogenous IL-2 does not rescue T-cell proliferation upon diflorasone treatment.
Freshly isolated T cells were treated with 1μM diflorasone2 hours and subsequently stimulated with CD3/28 immobilized on 96-well plate in the presence or absence of 100U/mL recombinant IL-2 as indicated. Proliferation was determined by [3H]-TdR incorporation assay. 1 donor was included in 3 independent experiments and shown as a single dot.
